# Bioactive compounds in extracts from short rotation willow shoots known as pharmaceuticals and experimental demonstration of biostimulation of maize plants by these chemical complexes

**DOI:** 10.3389/fpls.2025.1650824

**Published:** 2025-08-04

**Authors:** Zoltán Zombori, Gabriella Szalai, Kamirán Áron Hamow, Miklós Hóvári, László Sass, Györgyi Ferenc, Tibor Janda, Dénes Dudits

**Affiliations:** ^1^ Institute of Plant Biology, HUN-REN Biological Research Centre, Szeged, Hungary; ^2^ Department of Plant Physiology and Metabolomics, Agricultural Institute, HUN-REN Centre for Agricultural Research, Martonvásár, Hungary

**Keywords:** active phytobiological metabolites, LC-MS/MS, metabolomic pathways, *Salix* sp., seed priming, spraying

## Abstract

Extracts from willow bark or leaves were shown to contain effective plant biostimulants and pharmaceutical compounds. Considering the limitation in the use of bark raw materials on large scale, here we analyzed the stimulatory effects of extracts from various organs of willow (Salix viminalis L) plants grown as short rotation shrub willow. Metabolomic analysis of water extracts from different organs (leaves, meristems, stems) of two triploid genotypes (Rába and Maros) revealed presence of bioactive compounds. Quantity of phenylpropanoids, flavones, flavonols, anthocyanins, aminobenzoate degradants, plant hormones and stylbenes varied between organs and genotypes. Several of these bioactive compounds are known as pharmaceuticals. Here we carried out phytostimulatory tests by using various extracts for seed priming and foliar spraying of maize plants in greenhouse and field experiments. Both the digital imagining of maize plants and measurements of the plant height showed variable stimulation of growth. Chlorophyll fluorescence parameters indicated improved photosynthetic performance. Increased seed weight/ear and 1000 seed weight (17%) were detected after foliar spraying. The present study supports the extended application of bioactive phytocompounds by offering a novel raw material, the short rotation willow shoots as sources of bioactive compounds for use in agricultural practice and pharmaceutical industry.

## Graphical Abstract

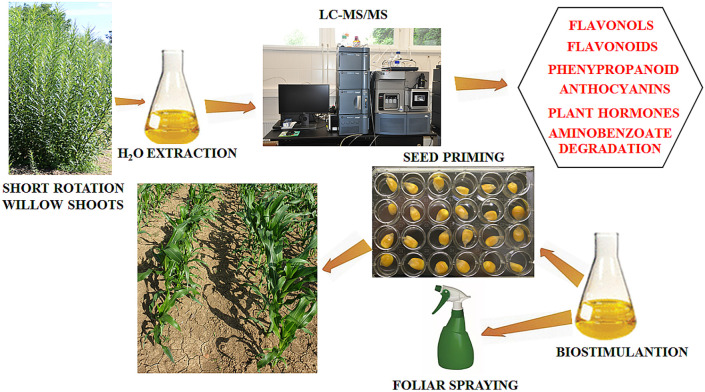

## 1 Introduction

Implementation of biostimulant strategies gain increasing attention not only in research but in agricultural applications (Han et al., 2024, Meena et al., 2025). According to du Jardin’s (2015) definition, natural plant biostimulator is the following: a plant biostimulant is any substance or microorganism applied to plants with the aim to enhance nutrition efficiency, abiotic stress tolerance and/or crop quality traits, regardless of its nutrients content. Baltazar et al. (2021) reviewed the broad range of biostimulants in crop cultivation, and molecular basis of their functionality. From this review one can conclude that the most widely used biostimulants are extracted from different seed weed species as *Ascophyllum nodosum, Ecklonia maxima*, and *Kappaphycus alvarezii*. According to Li et al. (2022), the overall results in the use of biostimulants in the agriculture are as follows: (1) among all biostimulant categories considering yield benefit is on average 17.9% and soil treatment is the most efficient, (2) biostimulant application in arid climates and vegetable cultivation had the highest impact on crop yield, and (3) biostimulants were more efficient in low soil organic matter content, non-neutral, saline, nutrient-insufficient, and sandy soils.

Several research has been reported about the positive effects of plant organ-derived chemicals on physiology and functions of plants (Mingzhao et al., 2024). Leaf extract of *Cupressus macrocarpa* (cypress) is an evergreen tree enhancing the antioxidation potential and photosynthetic capacity in zucchini under salt stress (ElSayed et al., 2022). Abd El-Mageed et al. (2017) reported that leaf extract of *Moringa oleifera* could improve drought tolerance in squash plants under saline conditions. *Ascophyllum nodosum* extract can alter the microbial community resulting an increase in root, shoot and fruit biomass parameters of pepper and tomato plants (Renaut et al., 2019). The root promoting biostimulant effects of willow bark extract was shown in chrysanthemum and lavender cuttings (Wise et al., 2020). Mutlu-Durak and Yildiz Kutman, 2021, 2023) reported that soaking/priming maize seeds in bark and leaf extracts of mature weeping willow tree (*Salix babylonica*) stimulated seedling shoot and root growth in the presence and absence of salinity stress. The willow bark extracts containing salicylic glycosides and salicylate can be used as fungicides (Marchand, 2016). Deniau et al. (2019); Gligorić et al., 2023 emphasized that willow extract (*Salix cortex*) is an important substance for agriculture.

In addition to using plant extracts as biostimulants, they provide economic sources for bioactive compounds with therapeutic potential (reviewed by Riaz et al., 2023). Preclinical and clinical studies proved the anti-inflammatory activity of salicylates derived from medicinal plants and willow bark (Tambo, 2023; Tienhao et al., 2021).

In the present study, we carried out metabolomic analysis of water extracts of organs from short rotation willow shoots to determine chemical composition of various extracts. Knowing the chemistry, we discuss published data about the roles of various compounds as potential crop biostimulants or pharmaceuticals. Furthermore, the experimental work is focused on testing whether one year old shoots of willow plants grown in short rotation system can serve as raw material for extraction of biostimulants. We provide data to compare the growth stimulatory effect of water extracts of leaves, meristems, stem from two short rotation willow genotypes. Effects of seed priming or foliar spraying maize plants were tested in green house by digital imaging of plant growth or under field conditions. To support sustainability of crop production, there is a demand for wider use of plant-derived stimulants in agricultural practice. Furthermore, the expanding “plant-based” pharmaceutical industry requires economic production of raw material. The present work provides a basis to consider short rotation willow plantations as source of bioactive compounds.

## 2 Materials and methods

### 2.1 Plant material and growth conditions in greenhouse and on field

This study utilized the Kiskun 4291 (FAO 290) hybrid maize cultivar provided by Kiskun Kutatóközpont Kft. Kiskunhalas, Hungary. In greenhouse experiments, the seeds were sown in radio-tagged Plexiglass columns encased in polyvinyl chloride tubing. The columns were filled with a soil mixture consisting of 80% peat (Florimo, Kecel, Hungary) and 20% sand, supplemented with 6 g of Osmocote fertilizer (Substral, Evergreen Garden Care, UK). Five seeds were seeded for each combination, and 2x10 seeds for the control group. The installed columns were arranged randomly within an automated modular phenotyping system (Photon System Instruments, Drasov, Czech Republic) and irrigated to maintain 60% field capacity. The growth temperature was sustained at 24°C during the day and 20°C at night, with relative humidity ranging between 60% and 80%. The illumination level was determined by the modular phenotyping system and set to 400 μmol photons m^-^² s^-^¹, with minor variations detected throughout the experiment.

In the field experiments, maize seeds were sown at two different locations: our experimental garden in Szeged, Hungary (46°14’01.1”N 20°10’01.1”E) and at the breeding garden of the Kiskun Kutatóközpont in Mórahalom, Hungary (46°10’48.2”N 19°54’09.2”E). The row spacing was 70 cm, and the plant spacing was 50 cm; the rows extended 8 m in Szeged and 10 m in Mórahalom. Two rows were implemented for each combination, accompanied by an additional four rows of control plants. The maize plants were not irrigated throughout the experiment, during the growing period precipitations were the following: Szeged: 78mm, Mórahalom:57mm.

### 2.2 Preparation of extracts from one year old willow shoots

The genotypes Maros and Rába of short rotation willows were used in the extraction processes. These genotypes originated from our triploid willow breeding program (Dudits et al., 2022). The willow plants were grown at our experimental garden in Szeged, Hungary (46°14’01.1”N 20°10’01.1”E). The plant material for the extraction was collected in May 2024. Around 80 cm long shoots were cut from the plants, and the stems, leaves and meristematic tissues were collected separately. 30g of these materials were finely chopped and placed into beakers on a heated magnetic stirrer containing 750 ml 90 °C distilled water. The plant tissues were extracted at this temperature for 30 min with continuous stirring. The extracts were cooled down and filtered, then stored at 4 °C or -20 °C for prolonged preservation.

### 2.3 Metabolomics of willow extracts

All solvents and chemicals used for sample preparation and analysis were UPLC gradient/GC pestinorm supratrace grade (VWR, Radnor, PA, USA). Willow extract samples were diluted with methanol: water (2:1 v/v%) solution to 0.5 ml/ml followed by vigorous vortex mixing and centrifugation at 16500 g (at 4°C for 10 min), and 1 ml of the methanol-water sample solution was liquid-liquid partitioned by adding 0.5 ml of n-hexane. Afterwards, centrifugation at 10000 g (at 4°C for 10 min) was addressed to collect the methanol: water phase that was finally filtered through 0.22 µm PTFE syringe filters prior to analysis. For analysis a Waters Acquity I-class UPLC system equipped with PDA detector were coupled to a Xevo TQ-XS Triple Quadrupole Mass Spectrometer with a UniSpray™ (US) ion source (Waters Corp.; Milford, MA, USA) was utilized. Separation of phenolic compounds was achieved on a HSS T3 column (1.8 μm; 100 mm × 2.1 mm) as described by Vrhovsek et al., 2012. Xevo TQ-XS MS was utilized in multiple reaction monitoring (MRM mode) according to Pál et al., 2020; UPLC and MS conditions and settings, method details, and respective MRM transitions used for quantification are listed in **Supplementary material Table S1**.

### 2.4 Methods of treatments with willow extracts

In seed priming experiments, maize seeds were immersed in water or the original willow extracts for one day before sowing in either a greenhouse or a field. In foliar spray experiments, the willow extracts were diluted 1:1 with distilled water supplemented with a non-ionic wetting and sticking agent, Nonit (Agrokemia Sellye, Sellye, Hungary) at a concentration of 0.0025%. The treatment was performed once during the field experiment when the plants were 5 weeks old. The foliar spray treatment in the greenhouse was carried out twice; on the 23^rd^ and 30^th^ day.

### 2.5 Digital phenotyping the growth of maize plants

Irrigation and digital imaging of the shoots were performed twice per week. Water consumption was continuously monitored, and irrigation was automatically adjusted by the phenotyping system. For imaging of the shoots, each column was transferred to an imaging chamber equipped with a rotatable platform, where two RGB cameras captured seven side-view and one top-view image of each maize shoot. Image segmentation and the extraction of shoot parameters were carried out automatically using the system’s software according to predefined settings.

Root imaging was conducted once per week. The Plexiglass columns were photographed from four side angles (90° apart) and from below using two Canon EOS 600D digital cameras in a separate imaging chamber located outside the PSI modular system, following the protocol described by Zombori et al. (2023). Root-associated white pixels were isolated by subtracting the background of the dark soil using custom-developed software. The visible root surface area was quantified by integrating data from the four side views and the bottom view.

### 2.6 Characterization of growth and biomass of maize plants in field conditions

The heights (cm) of maize plants developed from extracts or water-primed seeds were measured three times post-germination in the field experiment: at the developmental stage of three, five, and seven leaves. The plants subjected to foliar spray were measured prior to treatment and ten days after the treatment.

### 2.7 Chlorophyll fluorescence measurements

Chlorophyll fluorescence was measured using a Pocket PEA portable fluorimeter (Hansatech Instruments, King’s Lynn, UK). After a 20-min dark adaptation period, the minimal fluorescence yield (F_0_) was recorded. Leaves were subsequently exposed to a saturating light pulse of 3500 μmol m^-^² s^-^¹ for 1 s to determine the maximal fluorescence yield (F_m_) and the variable fluorescence (F_v_). Additionally, several chlorophyll fluorescence parameters were calculated using appropriate formulas and equations of the JIP-test, which yields multiple parameters quantifying the photochemistry of Photosystem II (Strasser et al., 2004).

### 2.8 Statistics

The data are shown as the mean of a minimum five replicates ± standard error of the mean. The statistical significance of the observed variations in different parameters was determined by using Student’s T-test *via* the online software GraphPad (www.graphpad.com). Statistical significance was defined at p ≤ 0.001 ***, p ≤ 0.01 **, and p ≤ 0.05 *.

## 3 Results and discussion

### 3.1 Genotype- and organ-dependent chemical composition of water extracts from short rotation willow shoots

Metabolites in water extracts from the leaves (L), stems (S), and meristems (M) of Rába (R) and Maros (M) genotypes were identified and quantified by LC-MS/MS in the following categories: 14 compounds in phenylpropanoid biosynthesis; 5 compounds in flavonoids; 4 compounds in flavones; 18 compounds in flavonols; 6 in anthocyanins; 10 in aminobenzoate degradation; 6 in plant hormones; 2 in stilbenes; and 1 in coumarins (see **Supplementary Table 1**). Here we give quantitative data about the identified compounds in different categories with potential bioactivity. Furthermore, we provide literature references discussing biostimulatory and therapeutic role of defined compounds detected in short rotation willow extracts. **Table 1**. presents selected compounds with considerable amount (ng/ml) in various willow extracts.

**Table 1:** List of selected chemical compounds with considerable amounts (ng/ml) in water extracts from various organs of two short rotation willow genotypes.

	Rába			Maros		
	Leaves	Meristem	Stem	Leaves	Meristem	Stem
Phenylpropanoid biosynthesis						
Total	12324.8	6264.77	1927.89	14882.5	7973.52	11356.5
coniferyl alcohol	142.73	264.67	349.33	96.93	145	107.33
salicin	51.2	37.6	880.67	72.87	1560	10393.3
neochlorogenic acid	1427.33	1425.33	154.47	3480	1560	213.87
chlorogenic acid	8206.67	3426.67	203.6	8380	3120	432
Flavonoids						
Total	119155	74673.8	16858.4	112552	61876.6	33004.8
phloridzin	824.67	521.33	184.47	937.33	482	258
Flavones						
apigenin-7-O-glucoside	10613.3	12866.7	127.87	9073.33	147.73	147.73
luteolin-7-O-glucoside	302.67	205.2	4.52	6720	3293.33	228.67
Flavonols						
kaempferol-3-O-glucuronide	3753.33	214.07	13.35	1263.33	82.73	2.71
quercetin-3-O-glucoside	5346.67	628	95.73	6186.67	550	43.53
quercetin-3-O-glucuronide	12173.3	1272.67	71.27	14566.7	1084.67	46.27
isorhamnetin-3-O-glucoside	10546.7	1160	112.33	10100	842.67	47.4
Anthocyanins						
procyanidin B1	10286.7	9006.67	2926.67	9726.67	6780	4386.67
procyanidin B3	19086.7	7793.33	2473.33	10706.7	5726.67	7920
cathecin	17613.3	17326.7	7000	13166.7	12920	10320
gallocathechin	14786.7	15666.7	1315.33	16833.3	13513.3	6053.33
Aminobenzoate degradation						
Total	239.25	168.46	634.52	139.78	1903.79	4184.89
4-hydroxybenzoic acid	33.73	16.73	148.87	19.26	10.21	14.84
3,4-dihydroxybenzoic acid	40.27	27.73	25.93	26.27	19.75	15.67
catechol	53.87	48.93	377.33	36.6	1835.33	4120
Plant hormones						
IAA - Indole3AA	0.38	3.5	1.66	0.33	2.22	0.3
jasmonic acid	73.67	12.73	12.73	122.27	53.93	23
abscisic acid (ABA)	1.68	7.71	7.71	1.88	14.85	1.27
phaseic acid (PA)	1.2	1.27	1.27	0.97	2.3	nd
dihydro-phaseic acid (DPA)	0.64	1.39	1.39	0.31	4.45	nd
Stylbenes						
cis-piceid	140.73	98.13	48.33	411.33	106.2	40.27

#### 3.1.1 Phenylpropanoids

Phenylpropanoids are bioactive secondary metabolites that significantly contribute to plant growth, development, and responses, including tolerance and resistance to abiotic and biotic stresses (Dong and Lin 2021). The total amounts of phenylpropanoids in extracts from all organs of the Maros (M) genotype were significantly higher than those in extracts from Rába (R) genotype. Leaf extracts generally contain more phenylpropanoids than stem and meristem extracts. The present analysis of chemical composition of willow extracts reveals a significant quantity of several compounds of phenylpropanoid metabolism with considerable quantity. **Table 1**. presents the selected ones:

Cinnamic acid (CA): Application *cis* -CA at 1-5 2.5 µM concentration to Arabidopsis roots stimulates both cell division and cell expansion in leaves, resulting in increased biomass (Steenackers et al., 2019). Concentrations of CA were higher in extracts from Rába meristem and stem compared to those from Maros genotype (**Supplementary Table 1**). Research indicates that cinnamic acid exhibit antioxidant, antimicrobial, anticancer neuroprotective, anti-inflammatory, and antidiabetic properties (Kumar and Parle, 2019).

Salicin and Salicylic acid: Salicin is a naturally occurring compound present in several plant species, including willow trees (Salix species), poplar trees (Populus species), and meadowsweet *(Filipendula ulmaria*). It is a glycoside, which means that it is a sugar molecule attached to a non-sugar molecule. In the instance of salicin, the non-sugar component is saligenin, a phenolic compound. Salicylic acid (2-hydroxy benzoic acid) is an important organic acid playing role in responses to both biotic and abiotic stress (Pál et al., 2020; Li et al., 2025).

In willow extracts used in the present study, salicin is present in higher amounts than salicylic acid concentrations (**Table 1**). Salicin was extracted in high amounts from the stems, particularly those of Maros genotype. Meristems of both genotypes contain more salicylic acid compared to leaves and stems. Positive effects of exogenously supplied salicylic acid in large number of plant functions have been demonstrated by several studies (Wollenweber, 2022). Salicylic acid influences physiological functions in a concentration-dependent manner, stimulating at low concentrations and inhibiting at high concentrations. Szalai et al. (2016) reported that priming maize seed with 0.5 mM salicylic acid solution for 1 day before sowing was beneficial during the early growth stage of the plants and might substantially enhance grain yield under natural field conditions. Salicylic acid has a crucial role in enhancing plant resistance to climate change related abiotic stresses, by facilitating interactions with other phytohormones, coordinating plant responses to stress, and utilizing several pathways to improve stress tolerance (see review by Elsisi et al., 2024). The analysis of the phytochemical contents of extracts from *Salix kochiana* showed lower concentration of salicylic acid (0.12 mg g^−1^) compared to salicin (2.11 mg g^−1^ (Lee et al., 2023). Similar differences were detected in the present study (**Table 1**). Extremely high salicin amount was found in the stems of Maros genotype. The bark extracts yielded 4300 ng/ml^-1^ salicin for *Salix alba* and 1167 ng/ml^-1^ of salicin for *Salix purpurea* using 90% vol. ethanol extraction (Carpa et al., 2022). Most research have primarily examined the effect of salicin in regulating plant growth through the use of complex extracts from willow bark or leaves, which contain several bioactive chemicals (Mutlu-Durak and Yildiz Kutman, 2021). Preclinical and clinical studies proved the anti-inflammatory activity of salicylates (Tambo, 2023).

Neochlorogenic acid (3-O-caffeoylquinic acid) is an isomer of chlorogenic acid (5-*O*-caffeoylquinic acid) resulting from the esterification of caffeic acid and D-(-)-quinic acid. Both are natural polyphenolic compounds. As shown by **Table 1** the quantities of these compounds in willow extracts are genotype- and organ dependent. The chlorogenic acid content is higher in the leaves of both genotypes. Regarding neochlorogenic acid content, Maros organs accumulate more than Rába shoot organs. Foliar sprays of *Lonicera japonica* plants, a traditional herb in East Asia, significantly enhanced both leaf length and width due to the presence of chlorogenic acid (Zhang et al., 2024). The biological activities of chlorogenic acid, primarily manifested as antioxidants, liver and kidney protection, antibacterial properties, antitumor effects, regulation of glucose and lipid metabolism, anti-inflammatory actions, neuroprotection, and effects on blood vessels, render its presence in willow extracts noteworthy. It can play an active role in safeguarding human health (Wang et al., 2022).

*p*-Coumaric acid (*p*-CA): Treatment of *Salvia hispanica* (chia) seedlings with *p*-CA resulted in enhanced growth, notably enhancing shoot length, fresh and dry weight (Nkomo et al., 2019) These authors reported significant rise in total chlorophyll (37.6%) and carotenoid (25.1%) content in response to exogenous application of *p*-CA. Meristem derived willow extracts contained higher amounts of *p*-coumaric acid in both genotypes (**Supplementary Table 1**). Antiproliferative, nephroprotective, neuroprotective, antioxidant and antimicrobial effects in addition to other biological properties of p-coumaric acid were shown by numerous *in vivo*, *in vitro* and clinical studies (Chen et al., 2024).

Gallic acid: Foliar applications of 20 µM gallic acid in combination with 5 μM zinc ferrite nanoparticles enhanced the growth, chlorophyll content, and gas exchange parameters of wheat subjected to salinity stress (Shao et al., 2024). The Rába stem extract contains the highest amount of gallic acid (**Supplementary Table 1**).

#### 3.1.2 Flavonoids

Flavonoids control physiology and phenotypic traits by acting as transcription regulators, influencing the auxin/cytokinin relationship and determining growth characteristics. Many plant flavonoids exhibit antioxidant activity, protecting plant tissues from harm caused by reactive oxygen species (ROS) or oxidative stress. Flavonoids thus protect plants from abiotic stresses as UV radiation, drought, salinity, and temperature fluctuations, as well as biotic stresses caused by insects, herbivores, nematodes, pathogens, and other organisms (see review by Patil et al., 2024). All these characteristics support the use of plant-derived flavonoids as biostimulants. As an example, the liquid suspension biostimulant MX42SEK contains bioflavonoids such as protocatechuic acid, quercetin, chlorogenic acid, coumaroyl quinic acid, and gentistic acid (Salvage et al., 2024). In the container experiment, this complex biostimulant enhanced photosynthetic ability, evidenced by elevated chlorophyll levels, greater specific leaf areas, and significantly bigger leaf assimilation areas. The treatment also significantly increased tuber yield by an average of 33% in tuber weight across three potato varieties and promoted the formation of larger-sized tubers.

##### 3.1.2.1 Flavonols

Daryanavard et al. (2023) provided a comprehensive review on the multifunctional roles of flavonols in plant biology. This includes alteration of plant hormone distribution that controls plant growth and development. Flavonols are scavengers of reactive oxygen species that can protect plants under environmental stresses as elevated temperature or drought. As shown by **Supplementary Table 1** the willow extracts are reached in flavonols. **Table 1** presents some of these compounds with considerable quantity in the form of flavonoid glycosides. In the present analysis, leaves of both maize genotypes have high concentration of glycolyzed form of flavonols. Differences in quantity of glycolyzed and aglycone forms is clearly visible in the case of quercetin. Quercetin shows the higher potential in terms of antioxidant property than quercetin-3-O-glucuronide. Here we must emphasize that flavonoid glycosides can be converted to their aglycone form by hydrolases (see review by Xiao et al., 2017). Spraying wheat seedlings with 3% of potassium quercetin solutions stimulated chlorophyll content and fluorescence and gas exchange and the total antioxidant capacity (Janczak-Pieniazek et al., 2021).

##### 3.1.2.2 Anthocyanins

Jan et al. (2024) reported that anthocyanin-treated rice plants exhibit improvements in different morphological and biochemical traits, including increased shoot and root length, chlorophyll content, relative water content, panicle number, and seed weight. Additionally, anthocyanin application reduces oxidative damage by lowering levels of reactive oxygen species, lipid peroxidation, and enhancing antioxidant enzyme activities. In the present study, the interpretation of biostimulant effect of willow extracts requires the consideration of high concentration of anthocyanins (see **Table 1**). These flavonoids were extracted with high amounts from leaves and meristems. Proanthocyanidins could prevent inhibitory effects of excess copper as plant growth and restoration of lignin synthesis is reported (Zhao et al., 2021).

#### 3.1.3 Plant hormones

As shown by **Table 1** willow extracts contain plant hormones as growth regulators, but relatively low concentration. They may degrade during extraction or storing procedure. Despite low concentration, these molecules can exhibit physiological effects. The amount of jasmonic acid is relatively high, especially in extracts from Maros organs.

### 3.2 Priming maize seeds and foliar spraying with extracts from stems or meristems can stimulate plant growth: greenhouse study

**Figure 1:**
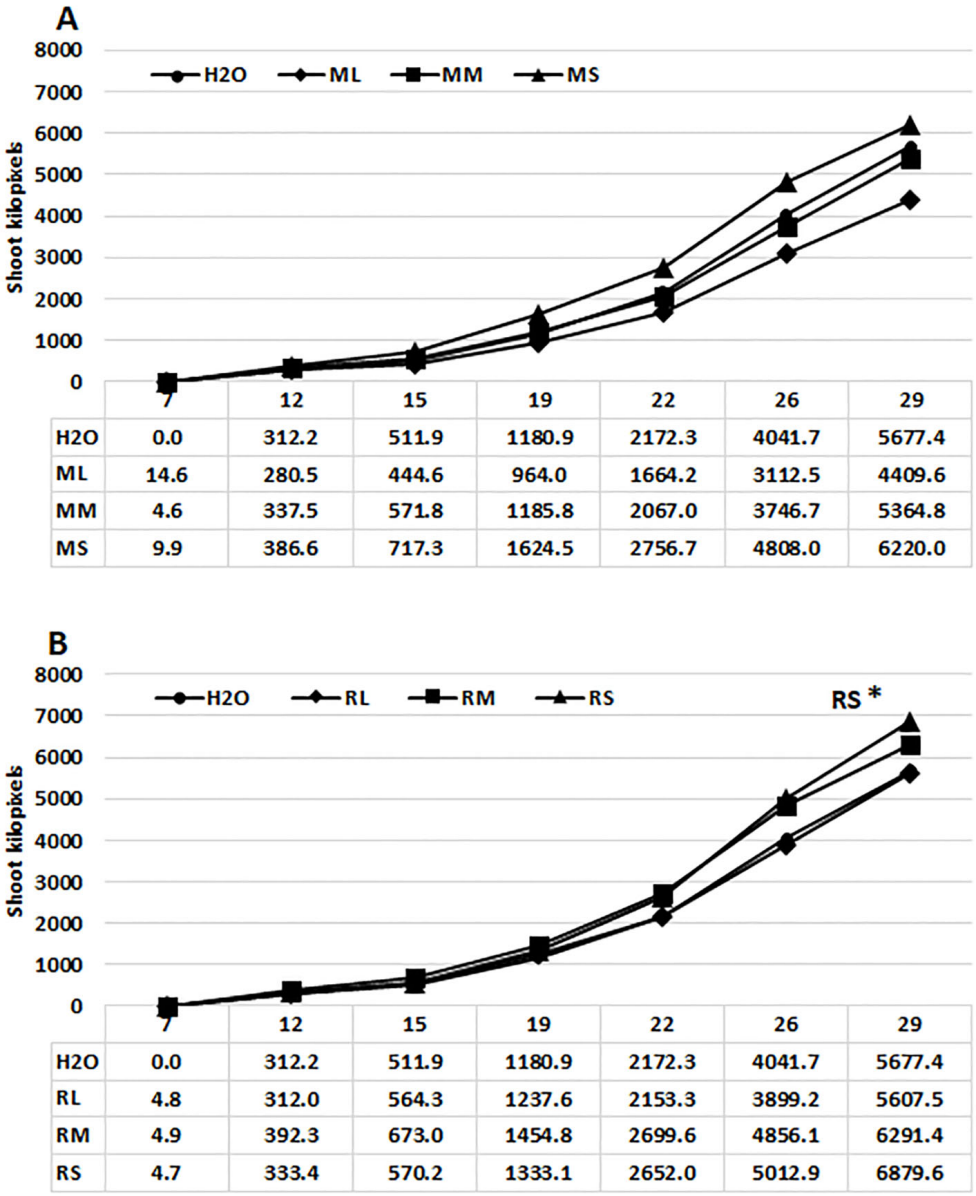
Effects of priming hybrid maize seeds with water extracts from willow plants on shoot surface area. **(A)** growth curve of maize plants primed with Maros extracts (total green pixels); **(B)** growth curve of maize plants primed with Rába extracts (total green pixels); All the parameters were quantified by digital imaging. ML, Maros leaf; MM, Maros meristem; MS, Maros stem; RL, Rába leaf; RM, Rába meristem; RS, Rába stem. Statistically significant events are denoted as p ≤ 0.05 *, N=5 plants.

Studies involving diverse plant species (Golzarian et al., 2011; Fehér-Juhász et al., 2014) indicate that green pixel values, which represent leaf and stem surface area are assumed to be directly proportional to the green biomass of plants. Consequently, this method was employed for comparing stimulatory effects of extracts derived from different organs of Rába and Maros willow genotypes on growth of maize plants. The phenotyping system employed in this work facilitated the cultivation of maize plants for 29 days under controlled optimal conditions (**Figures 1A, B**). Priming commercial maize hybrid seeds with water extracts from stems or meristems of willow plants can enhance shoot surface area and plant height. The stimulatory effect varied between extracts from the two genotypes and their respective organs. The mean shoot surface areas of maize plants were significantly greater following seed priming with Rába stem extract, in comparison to the plants treated with water or leaf extract (**Figures 1B**). The stimulatory effects of Maros and Rába stem extracts are expressed in the final growing period, as indicated by pixel values (**Figures 1A, B**). Extracts from Rába meristem tissues showed positive effect that did not attain statistical significance.

**Figure 2:**
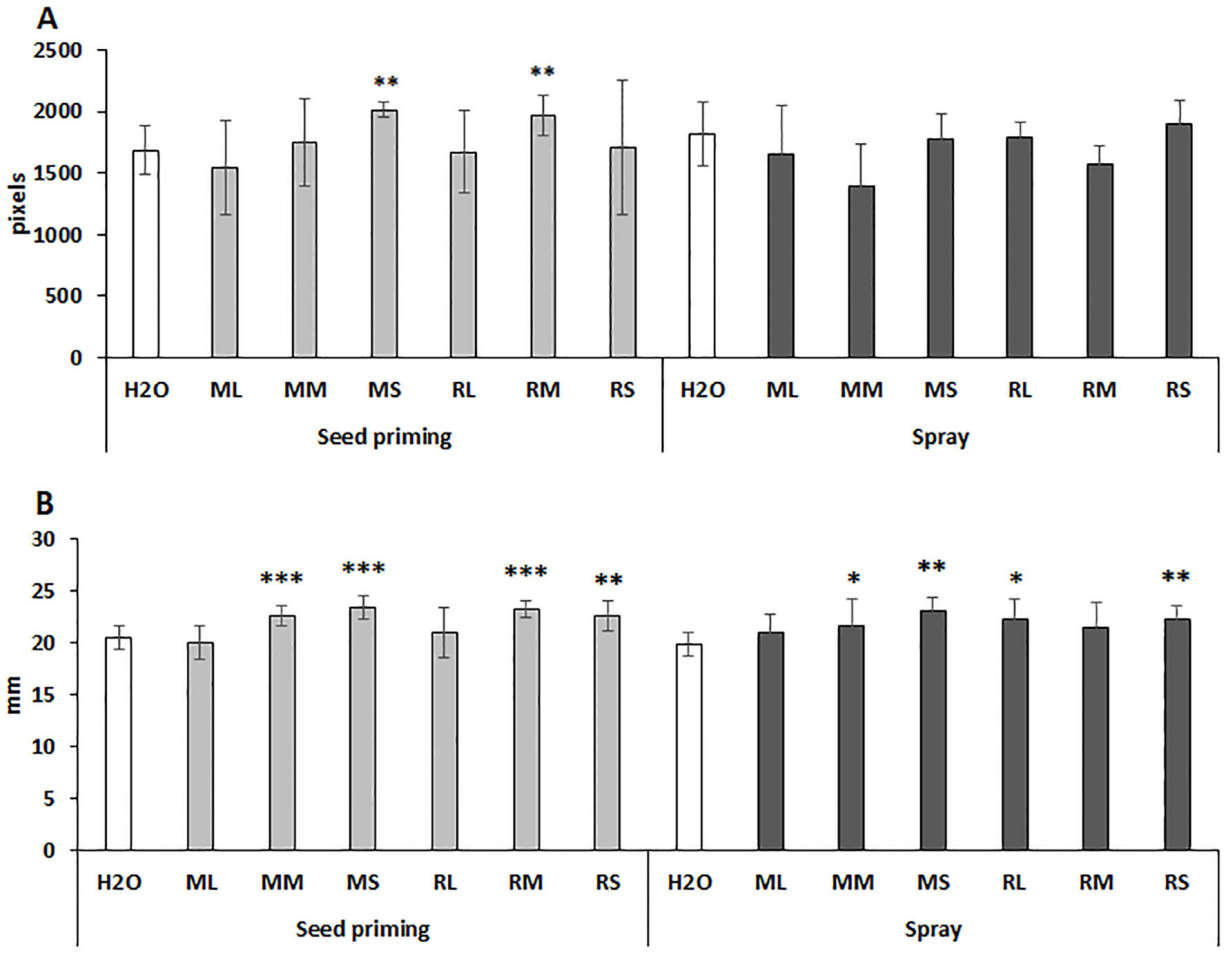
Differential response of plant height **(A)** and stem diameter **(B)** of maize plants to seed priming and foliar spraying with willow extracts. ML, Maros leaf; MM, Maros meristem; MS, Maros stem; RL, Rába leaf; RM, Rába meristem; RS, Rába stem. Statistically significant events are denoted as p ≤ 0.001 ***, p ≤ 0.01 **, and p ≤ 0.05 *, N=5 plants.

Physiological effects of willow extracts were also analyzed by determination of pixel-based plant height and stem width after spraying of maize plants at two time points: day 22nd and day 29th. As shown by **Figure 2A** seed priming with Maros stem and Rába meristem extracts significantly increased plant height. Spraying with Rába stem extract had a small but not significant positive effect on this parameter (**Figure 2A**). Interestingly, both priming seed and foliar spraying stimulated the width of maize stems (**Figure 2B**). Presently, we cannot explain the reason for differential responses of these two traits to the foliar treatment since the used extracts represent a complex chemical composition.

### 3.3 Effects of seed priming and foliar spraying on early growth rate of roots in maize plants: phenotyping in greenhouse

**Figure 3:**
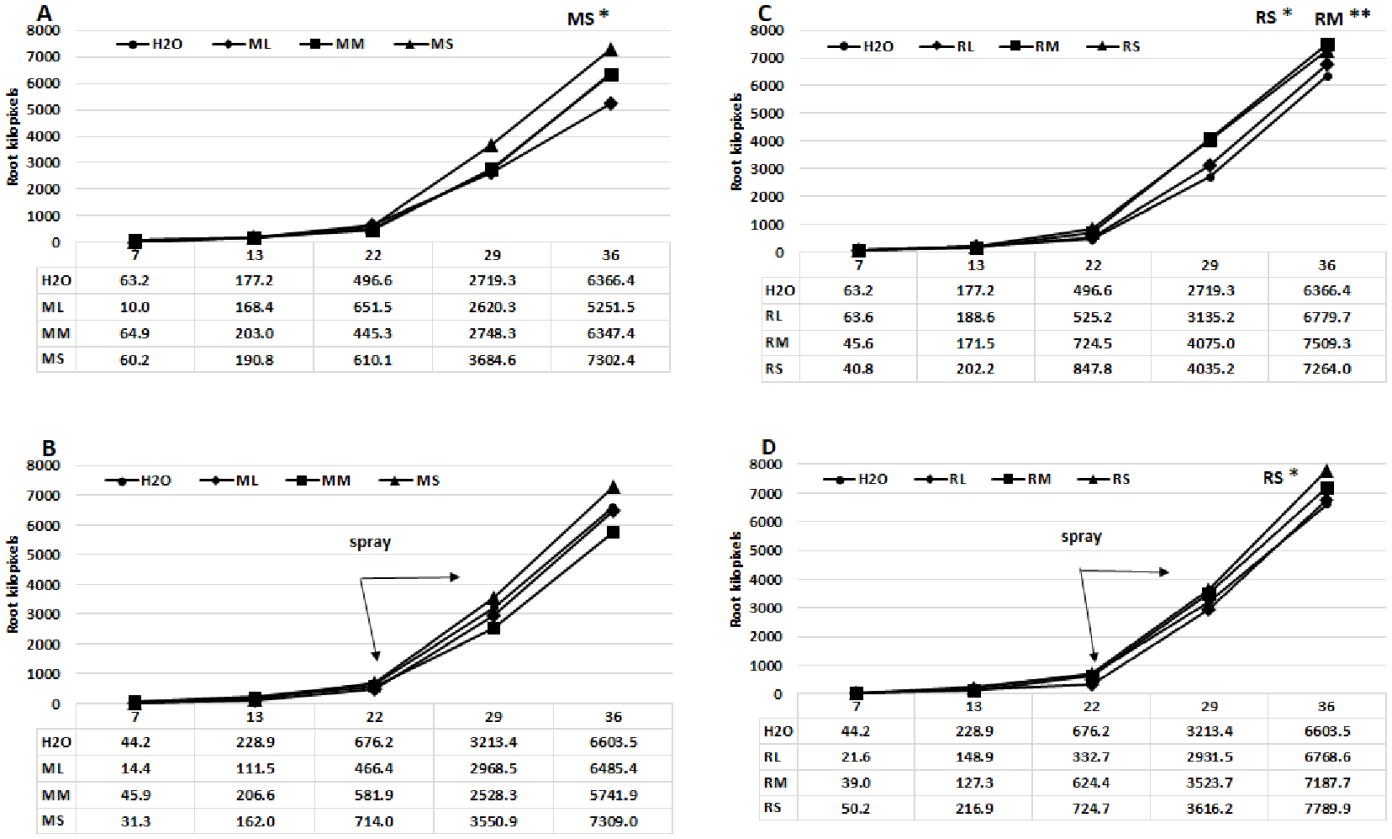
Growth curves of the root systems of hybrid maize plants treated with various willow extracts. **(A)** Seed priming with Maros extracts, **(B)** Foliar spray using Maros extracts, **(C)** Seed priming with Rába extracts, **(D)** Foliar spray using Rába extracts. ML, Maros leaf; MM, Maros meristem; MS, Maros stem; RL, Rába leaf; RM, Rába meristem; RS, Rába stem. Statistically significant events are denoted as p ≤ 0.01 **, and p ≤ 0.05 *, N=5 plants.

**Figure 4:**
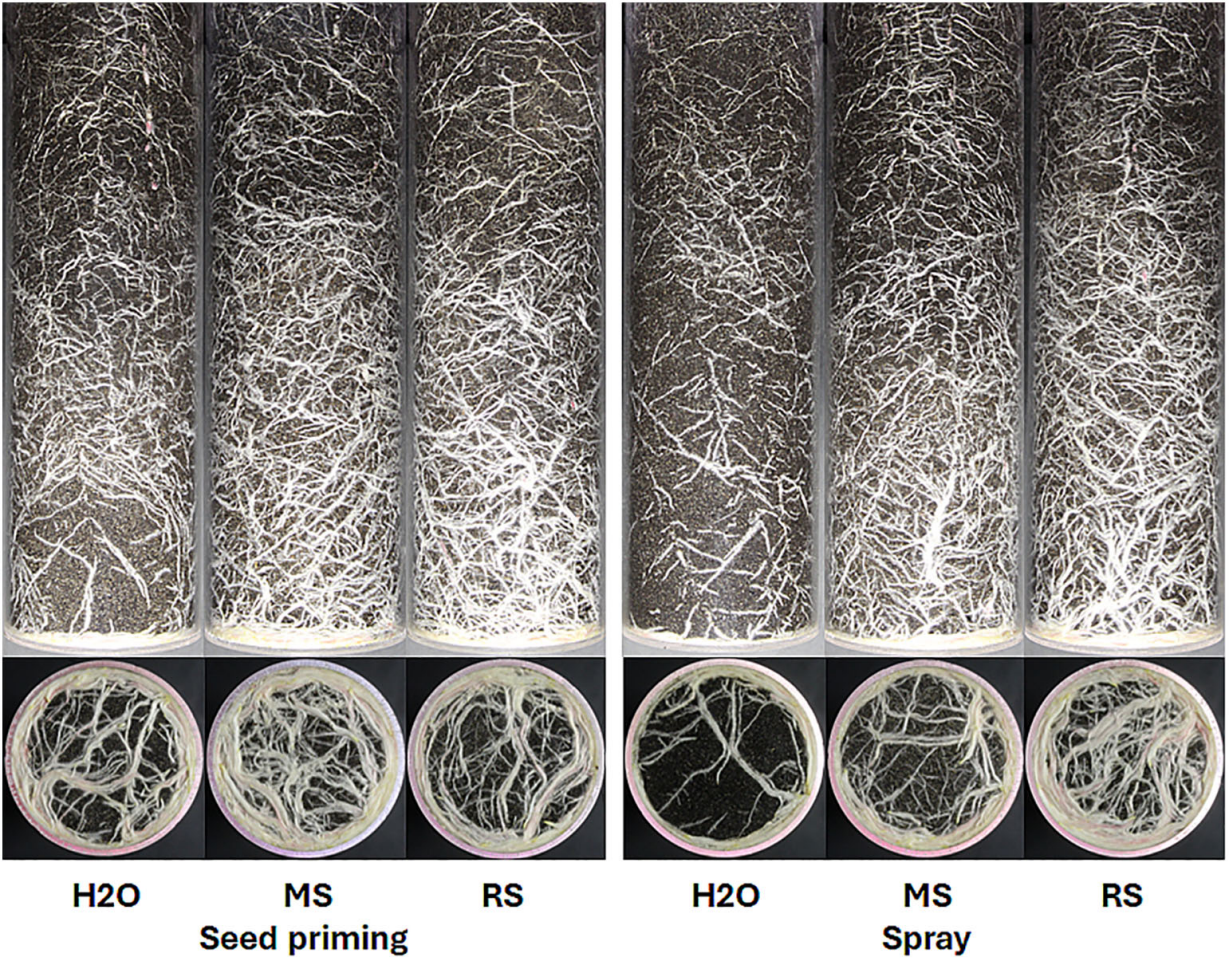
Stem extracts from two short rotation willow genotypes stimulate the development of root systems of maize hybrid plants following seed priming and foliar spray. MM, Maros meristem; MS, Maros stem; RM, Rába meristem; RS, Rába stem. Digital images were captured at the end of the growing period.

This study utilized a digital phenotyping system to ascertain the growth dynamics of the root system of the maize plants across various treatments. The growth diagrams (**Figures 3A–D**) indicate that alterations in root development are detectable after the third week. The seed priming treatment induced more significant changes, particularly with Rába meristem extract; however, the highest root pixel values were measured following the foliar spray treatment with Rába stem extract (7789.9 kilopixel, B). The stem and meristem extracts from shoots were generally more effective, regardless of genotype, except when using the Maros meristem extract in foliar spray treatment. **Figure 4** presents examples of characteristics of maize root systems under various treatments with extracts from Rába and Maros genotypes. Positive effects of biostimulators on root systems have been documented in several experiments (see review by Han et al., 2024).

### 3.4 Chlorophyll fluorescence parameters of maize leaves following willow extract treatment in greenhouse

In photosynthesis, a portion of the solar energy absorbed by plants is re-emitted as chlorophyll fluorescence (ChlF), which servs as an effective indication of the plant´s physiological condition in assessing plant growth responses (see review by Rinmawii et al., 2024). From the large set of ChlF parameters (Makhtoum et al., 2023), **Table 2** illustrates selective instances showing elevated values in maize leaves treated with various short rotation willow extracts. The examination of maize plants derived from treated seeds reveals a lasting impact, whereas ChlF markers detected in leaves one-week post-spraying signify an initial reaction. This difference may elucidate the finding that the set of responding individuals is fundamentally distinct between the two treatment methods. Moreover, the enhanced efficacy of Rába stem extract was detectable following seed treatment, although a comparable trend could not be seen after spraying.

**Table 2:** Effect of seed priming and foliar spray with willow extracts on Chl fluorescence defined by JIP parameters in maize leaves.

	SEED PRIMING			FOLIAR SPRAYING			
EXTRACTS	H_2_O	Maros Stem	Rába Stem	H_2_O	Maros Stem	Rába meristem	Rába Stem
FLUORESCENCE PARAMETERS							
**Sm**	33.74	34.34	37.34 *	41.95	39.64	41.67	41.14
**ABS/RC**	3.716	3.814	4.021 **	3.982	3.843	3.900	3.739
**N**	93.71	97.18	112.5 **	124.58	115.23	124.41	114.43
**PI/ABS**	1.876	1.830	1.962 *	1.888	1.986	1.918	1.862
**ETo/RC**	1.454	1.479	1.624 ***	1.577	1.554	1.550	1.457
**ψo**	0.703	0.707	0.722 **	0.716	0.713	0.712	0.704
**Fv/Fo**	2.936	2.850	2.965	2.879	3.046	2.948	2.913
**Fo**	5367	5454	5327	5357	5608	5559	5631*
**Fm**	21128	20996	21129	20766	22705 *	21947	22030
**RC/CSo**	1447.6	1432.7	1328 *	1356.9	1476	1480.0	1510.4 *
**RC/CSm**	5702.2	5509.6	5270	5242.2	5985 *	5633.1	5911.4 *

Selected chlorophyll fluorescence (ChlF) parameters indicate stimulation of photosynthetic functions by short rotation willow extracts from various organs.

Statistically significant events are in bold and denoted as p ≤ 0.001 ***, p ≤ 0.01 **, and p ≤ 0.05 *, N=5 plants.

**Figure 5:**
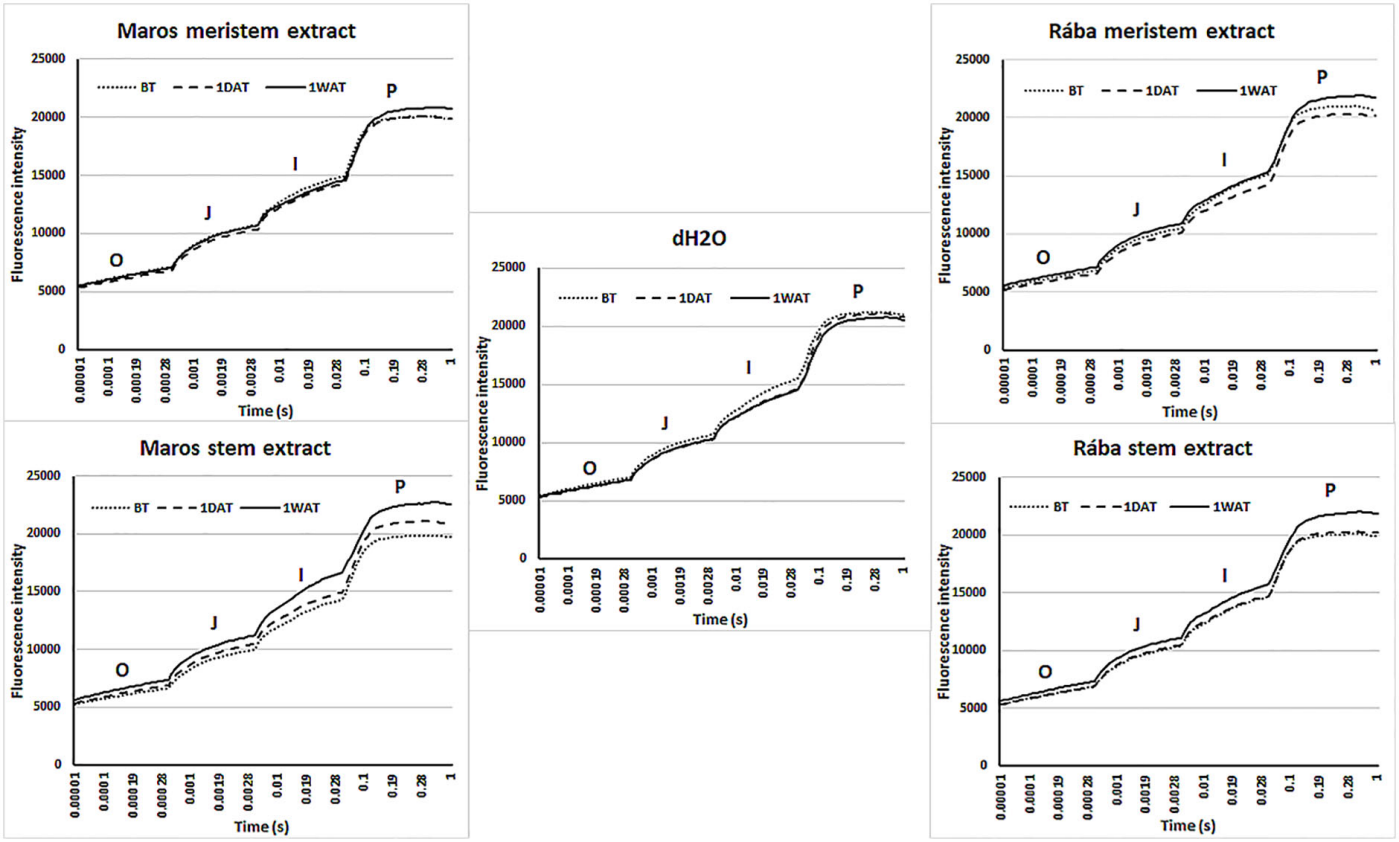
Kinetic representation of the OJIP transients from hybrid maize leaves that were treated with water or willow extracts derived from meristem and stem tissues. The kinetic curves of OJIP can indicate alterations in the primary photochemical reaction and the photosynthetic function of PSII. N=5 plants.

**Figure 5** displays kinetic curves of OJIP, which denotes “O” (minimum fluorescence), “J” (fluorescence after short light exposure), “I” (fluorescence after a longer light exposure), and “P” (maximum fluorescence) in maize leaves treated with water or willow extracts derived from meristem and stem tissues. These O-J-I-P transients show observed variations in the efficiency of the chlorophyll antenna in harnessing light energy, with the transfer to plastoquinone A (Q_A_, the primary electron acceptor) being the only limitation of photochemical conversion in PSII. Leaves treated with Maros stem extract caused a significant increase of ChlF during the “P” phase, particularly one week after foliar spraying. A slight increase is visible after one day.

As shown by **Table 2**, in maize leaves derived from primed seeds the following ChlF parameters show enhanced photosynthetic efficiency: Sm (Normalised Area; Area/Fv), which is directly proportional to the number of times the single QA molecule is reduced and oxidized (the primary quinone acceptor of photosystem II) during the fast OJIP transient, or the number of electrons passing through the electron transport chain; ABS/RC, the absorption flux per reaction center exciting PSII antenna Chl a molecules; N, the turnover number representing the frequency of QA reduction and re-oxidation; PI/ABS, the performance index for energy conservation from photons absorbed by PSII to the reduction of intersystem electron acceptors; ETo/RC, the electron transport flux (beyond Q_A_^−^) per reaction center; ψo, the maximum quantum yield for primary photochemistry (**Table 2**). Extract from Rába stems resulted in greater increase in ChlF values in maize leaves developed from primed seeds.

Different ChlF parameters showed increased chlorophyll fluorescence than in leaves after spraying with effective extract: Fo: minimum fluorescence, measured in dark adapted state, when all PSII RCs are open (≅ to the minimal reliable recorded fluorescence); Fv: maximal variable fluorescence, measured in dark adapted state; Fm: maximum fluorescence, measured in dark adapted state, when all PSII RCs are closed; RC/CSo: Density of reaction centers per excited represents cross section; Fv/Fm·(VJ/Mo)·Fo, where VJ is the relative variable fluorescence at J step, and Mo is the initial slope of the relative variable fluorescence): represents the ratio of active reaction centers to the total number of reaction centers; RC/CSm: performance index for energy conservation from photons absorbed by PSII until the reduction of intersystem electron acceptors, represents the ratio of active reaction centers to Sm related active center; PI/ABS values are increased also after spraying with Maros stem extract. The use of extract from Rába stems to maize leaves proved particularly beneficial.

### 3.5 Plant height of maize plants developed from seeds treated with the extracts: field studies

**Figure 6:**
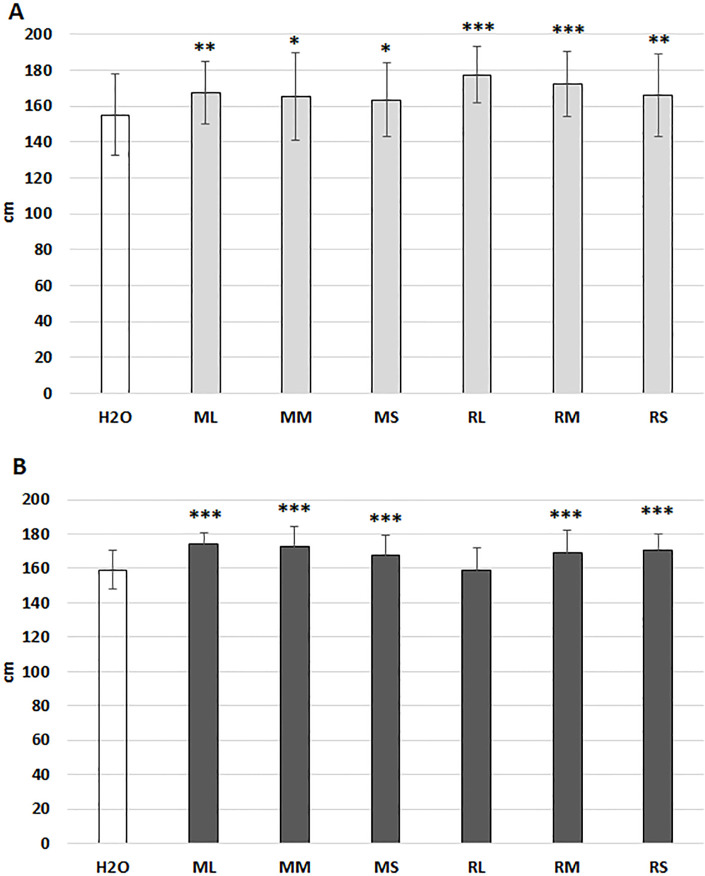
Height (cm) of maize hybrid plants at the seven-leaf stage as markers of growth characteristics in response to seed priming using various willow extracts. **(A)** measurements in the Szeged location; **(B)** measurements in the Mórahalom location. ML, Maros leaf; MM, Maros meristem; MS, Maros stem; RL, Rába leaf; RM, Rába meristem; RS, Rába stem. Statistically significant events are denoted as p ≤ 0.001 ***, p ≤ 0.01 **, and p ≤ 0.05 *, N=60 plants.

Digital phenotyping indicates moderated responses of maize plants to various extracts from short rotation willow plants in greenhouse with optimal growing conditions. Plant extract gains increasing significance as biostimulants (Han et al., 2024), therefore, we have evaluated various extracts under limited water supply and various soil conditions at two field study locations (Szeged and Mórahalom). For demonstrating the stimulatory effects of different short rotation willow extracts, we present plant height data (cm) in **Figure 6**. All extract treatments of maize seeds significantly stimulated plant growth at both locations. For unclear reason, extracts from Rába leaves exhibited significant activity in Szeged (**Figure 6A**), although no stimulatory effect was seen in Mórahalom location (**Figure 6B**). Conversely, leaf extracts from the Maros genotype exhibited significant activity.

### 3.6 Effects of foliar spraying on the growth of maize plants in the field

**Figure 7:**
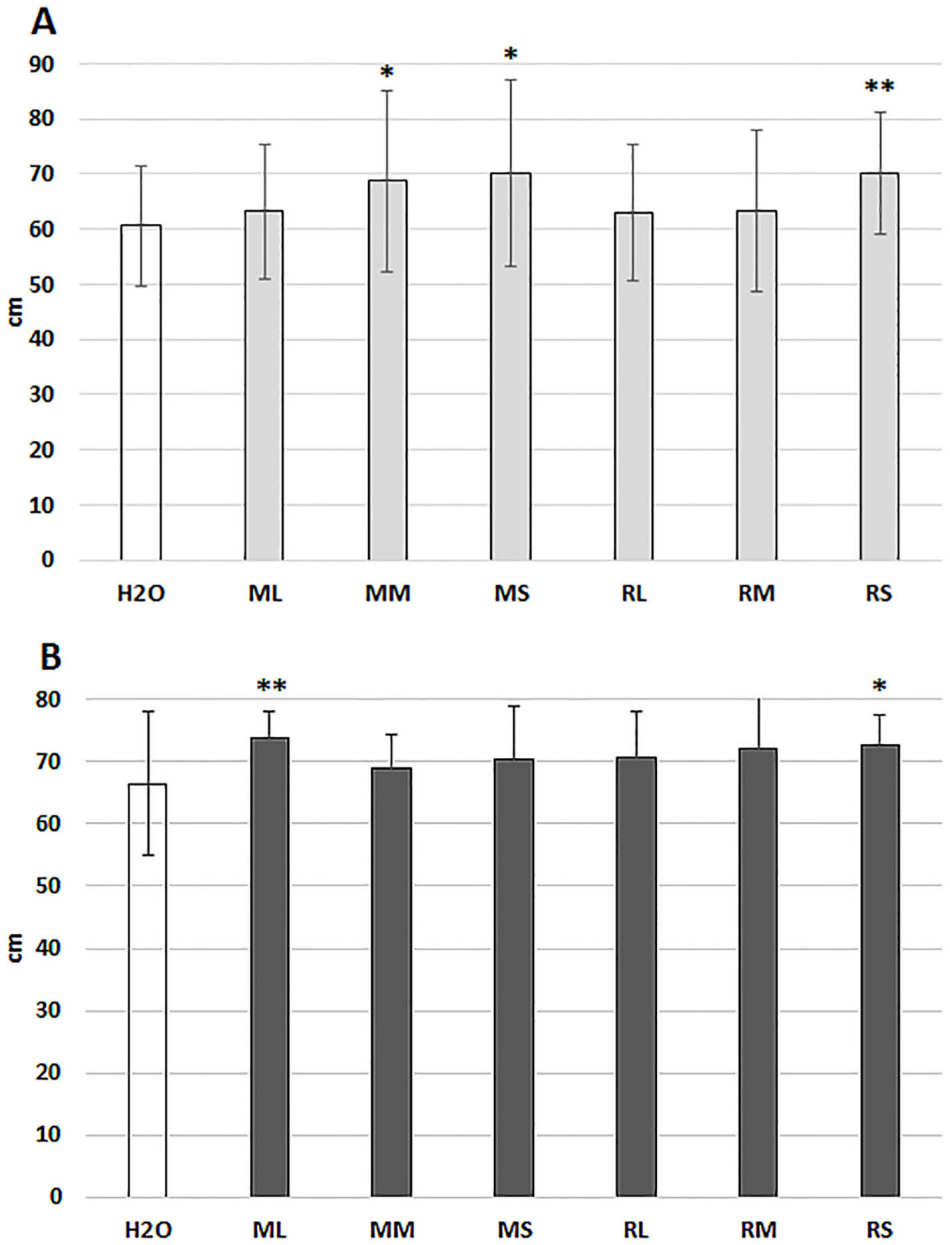
10-days growth of the hybrid maize plants following foliar spray with different willow extracts. **(A)** measurements in the Szeged location; **(B)** measurements in the Mórahalom location. ML, Maros leaf; MM, Maros meristem; MS, Maros stem; RL, Rába leaf; RM, Rába meristem; RS, Rába stem. Statistically significant events are denoted as, p ≤ 0.01 **, and p ≤ 0.05 *, N=60 plants.

Alongside seed priming, short rotation willow extracts were evaluated by spraying treatments (**Figures 7A, B**). Extracts from Rába stem tissues could induce significant growth stimulation in both locations. At Szeged experimental station, meristem and stem extracts from Maros genotype enhanced the growth of maize plants.

### 3.7 Alteration of seed yield parameters after foliar spraying

In addition to monitoring the stimulation of vegetative growth of maize plants, we have characterized seed yield parameters under field conditions in Szeged region. Part of the experimental field in Mórahalom was accidently irrigated, therefore we could not use these information. Primed seed-derived plants did not show significant changes in seed parameters compared to untreated plants. Spraying maize plants at the seven-leaf stage could influence seed weight (g) per ear and 1000-seed weight (g) in the selected genotype and organ extract treatments (**Table 3**). Based on seed parameters of untreated plants, we had to realize the variations in growing conditions in various parts of experimental field. Therefore, we use two datasets from plot A and plot B. Under ideal conditions (plot A), spraying with Rába meristem extract resulted in 12,10% increase in seed weight per ear. The stimulation of 1000-grain weight was 7,00%. In plot B, the Rába leaf extract increased seed weight per year by 17,42%, the Rába meristem extract by 16,79%, and the Maros stem extract by 12,99%. The weights of 1000 seeds were also stimulated by the Maros meristem extract (21.22%) and by Rába leaf extract (17,31%). These preliminary data show the same tendency as that was published by Li et al. (2022) after meta-analysis of yield stimulation. Furthermore, both the greenhouse study conducted under optimal conditions, and the present field results across various soil conditions agree with previous observations that the biostimulant impact can be pronounced under stress situations (Li et al. (2022).

**Table 3:** Grain yield parameters after foliar spray treatment in field experiments.

Area	Spraying solution	Seed weight			
		per ear		1000 grains	
		Weight	%	Weight	%
A	H2O	105.8 ± 8.4		214	
	RS	103.4 ± 20.9	97.7	216.5	101.1682
	RM	118.6 ± 14.4	112.1	229	107.0093
	ML	104.1 ± 13.7	98.4	215	100.4673
	MM	100.4 ± 14	94.9	206	96.26168
B	H2O	82.6 ± 12.2		179	
	RM	96.5 ± 13.8 *	116.8	207	115.6425
	RL	97 ± 7.9	117.4	210	117.3184
	MM	91.1 ± 13.6	110.3	217	121.2291
	MS	93.4 ± 27.9	113.1	190	106.1453
	ML	70.2 ± 15.9	85.0	199	111.1732

ML, Maros leaf; MM, Maros meristem; MS, Maros stem; RL, Rába leaf; RM, Rába meristem; RS, Rába stem. Szeged location; A and B plot.

Statistically significant events are in bold and denoted as p ≤ 0.05*, N=5 ears.

The foliar application of biostimulants is a cost-effective method strategy that is prioritized in crop production, particularly using seaweed extracts (Hariharan et al., 2024). In the present experiments, both in the greenhouse and the field, seed priming proved more effective than foliar spray for biomass production, although foliar spray was superior for yield increase. The seaweed extracts resulted in 17.1% enhancement. In our case, the overall stimulation by willow extract in yield was 13% as the maize plants productivity is concerned.

## 4 Conclusions

Previous studies demonstrated that extracts from bark or leaves of mature willow trees contain various biologically active compounds with potential in pharmaceutical or plant biostimulant applications. This study presents experimental data that expands the source of bioactive compounds using willow raw material as one-year-old shoots from willow plants cultivated in a short rotation system. The economic, large-scale production of willow raw material opens new potential for the broader use of willow biostimulant in crop cultivation systems. Our data show that water extracts from the above-ground organs of outgrowing shoots have stimulatory effect; therefore, whole cuttings can be used for extraction without the necessity for separate tissue collection. The variations in genotypes regarding efficacy highlight the importance of breeding for improved chemical composition. The currently evaluated Rába and Maros genotypes originate from a triploid hybridization program. Although seed priming can enhance the initial growth of maize plants, foliar spray technology has numerous advantages, as it can be integrated well with other cultivation methods and can facilitate improvements in seed production. The current metabolomic investigation indicates that short rotation willow shoots are abundant in several biologically active chemicals with recognized health benefits, thus holding potential for the pharmaceutical business. In the future, we intend to optimize extraction methods and treatments for crop plants.

## Data Availability

The original contributions presented in the study are included in the article/**Supplementary Material**. Further inquiries can be directed to the corresponding author/s.

## Supplementary material

Supplementary Table 1 Metabolites detected by LC/MS/MS analysis in water extracts from various organs (L, leaves; M, meristems; S, stems) of two short rotation willow genotypes (R, Rába; M, Maros).
