# Supplementary material for: Bioactive compounds in extracts from short rotation willow shoots known as pharmaceuticals and experimental demonstration of biostimulation of maize plants by these chemical complexes
Source: Front Plant Sci. 2025 Aug 4;16:1650824. doi: 10.3389/fpls.2025.1650824 (PMC12358415; doi:10.3389/fpls.2025.1650824)
Supplement: Supplementary Table 1 — Metabolites detected by LC/MS/MS analysis in water extracts from various organs (L, leaves; M, meristems; S, stems) of two short rotation willow genotypes (R, Rába; M, Maros). [file Table1.docx]

| **ng/ml** | **Phenylpropanoid biosynthesis** |  |  |  |  |  |  |
| --- | --- | --- | --- | --- | --- | --- | --- |
|  |  | **RL** | **RM** | **RS** | **ML** | **MM** | **MS** |
|  | **cinnamic acid** | 6,63 | 13,35 | 26,53 | 3,48 | 9,23 | 3,65 |
|  | **coniferyl alcohol** | 142,73 | 264,67 | 349,33 | 96,93 | 145,00 | 107,33 |
|  | **p-coumaric acid** | 62,27 | 121,67 | 18,25 | 54,33 | 114,80 | 21,48 |
|  | **gallic acid** | 27,53 | 41,33 | 95,13 | 33,87 | 28,00 | 33,73 |
|  | **methyl gallate** | 3,11 | 5,31 | 68,67 | 3,62 | 5,19 | 12,78 |
|  | **caffeic acid** | 60,33 | 42,00 | 14,31 | 41,20 | 34,27 | 6,75 |
|  | **ferulic acid 1 - trans** | 35,53 | 26,33 | 26,60 | 19,30 | 14,54 | 9,29 |
|  | **ferulic acid 3 - isomer** | 18,11 | 6,24 | 6,11 | 7,35 | 3,09 | 3,02 |
|  | **salicylic acid** | 19,32 | 23,60 | 8,35 | 11,57 | 29,40 | 5,64 |
|  | **salicin** | 51,20 | 37,60 | 880,67 | 72,87 | 2065,33 | 10393,33 |
|  | **neochlorogenic acid** | 1427,33 | 1425,33 | 154,47 | 3480,00 | 1560,00 | 213,87 |
|  | **cryptochlorogenic acid** | 654,67 | 526,67 | 57,67 | 907,33 | 578,67 | 72,27 |
|  | **chlorogenic acid** | 8206,67 | 3426,67 | 203,60 | 8380,00 | 3120,00 | 432,00 |
|  | **chlorogenic acid related unknown isomer** | 1609,33 | 304,00 | 18,20 | 1770,67 | 266,00 | 41,40 |
|  |  |  |  |  |  |  |  |
|  | **Flavonoids** |  |  |  |  |  |  |
|  |  | **RL** | **RM** | **RS** | **ML** | **MM** | **MS** |
|  | **robinin** | 299,33 | 50,73 | nd | 74,27 | 21,02 | nd |
|  | **phloretin** | 1,43 | 1,35 | 0,37 | 1,50 | 2,69 | 5,32 |
|  | **phloridzin** | 824,67 | 521,33 | 184,47 | 937,33 | 482,00 | 258,00 |
|  | **trilobatin** | 894,00 | 530,00 | 185,27 | 1058,67 | 528,00 | 266,67 |
|  | **naringenin** | 32,73 | 163,53 | 18,36 | 33,73 | 505,33 | 658,67 |
|  | **sakuranetin** | nd | 647,33 | nd | nd | 198,60 | nd |
| **Flavones** | **apigenin** | 141,13 | 39,33 | nd | 150,27 | 27,53 | nd |
|  | **apigenin-7-O-glucoside** | 10613,33 | 12866,67 | 127,87 | 9073,33 | 8713,33 | 147,73 |
|  | **luteolin** | 3,90 | 2,37 | 0,22 | 36,53 | 17,51 | 1,99 |
|  | **luteolin-7-O-glucoside** | 302,67 | 205,20 | 4,52 | 6720,00 | 3293,33 | 228,67 |
| **Flavonols** | **dihydro kaempferol** | 6,89 | 10,25 | 1,00 | 1,22 | 3,35 | 1,72 |
|  | **kaempferol-3-O-glucuronide** | 3753,33 | 214,07 | 13,35 | 1263,33 | 82,73 | 2,71 |
|  | **kaempferol-3-O-rutinoside** | 890,67 | 139,53 | 10,98 | 136,53 | 20,25 | 2,87 |
|  | **taxifolin** | 40,73 | 40,87 | 524,67 | 19,31 | 64,07 | 480,00 |
|  | **quercetin** | 119,07 | 9,42 | 6,13 | 60,87 | 8,55 | 18,51 |
|  | **quercetin-3-O-rhamnoside** | 2,23 | 0,52 | 3,43 | 1,29 | 0,65 | 2,68 |
|  | **quercetin-3-O-glucoside** | 5346,67 | 628,00 | 95,73 | 6186,67 | 550,00 | 43,53 |
|  | **quercetin-3-O-galactoside** | 114,20 | 28,80 | 4,12 | 86,27 | 8,16 | 4,34 |
|  | **quercetin-3-O-glucuronide** | 12173,33 | 1272,67 | 71,27 | 14566,67 | 1084,67 | 46,27 |
|  | **quercetin-3-Glc-Ara** | 7,01 | nd | nd | 710,00 | 24,60 | 81,73 |
|  | **quercetin-3,4'-diglucoside** | 154,67 | nd | nd | 125,07 | nd | nd |
|  | **isorhamnetin** | 72,07 | 4,05 | nd | 34,20 | 2,28 | nd |
|  | **isorhamnetin-3-O-glucoside** | 10546,67 | 1160,00 | 112,33 | 10100,00 | 842,67 | 47,40 |
|  | **isorhamnetin-3-rutinoside** | 2860,00 | 576,00 | 32,87 | 1432,00 | 109,93 | 11,68 |
|  | **rutin (quercetin-Glc-Rha)** | 3346,67 | 822,67 | 97,93 | 2593,33 | 244,67 | 45,93 |
|  | **myricetin** | 390,67 | 79,80 | 70,20 | 290,67 | 284,00 | 324,67 |
|  | **syringetin-3-O-glucoside + syringetin-3-O-galactoside** | 2133,33 | 564,67 | 35,33 | 5100,00 | 1558,00 | 51,00 |
| **Anthocyanins** | **procyanidin B1** | 10286,67 | 9006,67 | 2926,67 | 9726,67 | 6780,00 | 4386,67 |
|  | **procyanidin B3** | 19086,67 | 7793,33 | 2473,33 | 10706,67 | 5726,67 | 7920,00 |
|  | **epicathecin** | 1481,33 | 2640,00 | 1524,00 | 724,67 | 2600,00 | 1197,33 |
|  | **cathecin** | 17613,33 | 17326,67 | 7000,00 | 13166,67 | 12920,00 | 10320,00 |
|  | **gallocathechin** | 14786,67 | 15666,67 | 1315,33 | 16833,33 | 13513,33 | 6053,33 |
|  | **epigallocatechin** | 829,33 | 1661,33 | 18,69 | 601,33 | 1658,67 | 395,33 |
|  |  |  |  |  |  |  |  |
|  | **Aminobenzoate degradation** |  |  |  |  |  |  |
|  |  | **RL** | **RM** | **RS** | **ML** | **MM** | **MS** |
|  | **4-hydroxybenzoic acid** | 33,73 | 16,73 | 148,87 | 19,26 | 10,21 | 14,84 |
|  | **2,5-dihydroxybenzoic acid** | 3,45 | 0,84 | 0,67 | 1,35 | 0,79 | 0,38 |
|  | **2,6-dihydroxybenzoic acid** | 16,77 | 22,27 | nd | 6,13 | 12,59 | 0,00 |
|  | **3,4-dihydroxybenzoic acid** | 40,27 | 27,73 | 25,93 | 26,27 | 19,75 | 15,67 |
|  | **vanillin** | 37,93 | 14,58 | 26,80 | 19,77 | 8,83 | 14,71 |
|  | **acetovanillone** | 3,41 | 2,14 | nd | 1,18 | 0,75 | nd |
|  | **vanillic acid** | 29,27 | 19,04 | 11,05 | 15,98 | 7,42 | 3,89 |
|  | **syringaldehyde** | 14,34 | 10,53 | 40,33 | 7,61 | 5,27 | 13,72 |
|  | **syringic acid** | 6,21 | 5,67 | 3,54 | 5,63 | 2,85 | 1,68 |
|  | **catechol** | 53,87 | 48,93 | 377,33 | 36,60 | 1835,33 | 4120,00 |
|  |  |  |  |  |  |  |  |
|  | **Plant hormones** |  |  |  |  |  |  |
|  |  | **RL** | **RM** | **RS** | **ML** | **MM** | **MS** |
|  | **IAA - Indole3AA** | 0,38 | 3,50 | 1,66 | 0,33 | 2,22 | 0,30 |
|  | **jasmonic acid** | 73,67 | 112,00 | 12,73 | 122,27 | 53,93 | 23,00 |
|  | **jasmonic-acid-LE/ILE SUM** | 1,40 | 1,30 | 0,21 | 4,81 | 0,96 | 0,26 |
|  | **abscisic acid (ABA)** | 1,68 | 41,13 | 7,71 | 1,88 | 14,85 | 1,27 |
|  | **phaseic acid (PA)** | 1,20 | 6,96 | 1,27 | 0,97 | 2,30 | nd |
|  | **dihydro-phaseic acid (DPA)** | 0,64 | 16,91 | 1,39 | 0,31 | 4,45 | nd |
|  |  |  |  |  |  |  |  |
|  |  | **RL** | **RM** | **RS** | **ML** | **MM** | **MS** |
| **Stylbenes** | **trans-piceid** | 19,45 | 86,53 | 21,53 | 82,47 | 75,80 | 26,20 |
|  | **cis-piceid** | 140,73 | 98,13 | 48,33 | 411,33 | 106,20 | 40,27 |
| **Coumarins** | **esculin** | 15,49 | 1,77 | 0,85 | 14,53 | 0,90 | 0,34 |
